# Effect of dietary approaches to stop hypertension, and standard diets with and without curcumin on interleukin-1 alpha, 5-alpha reductase gene expressions, and androgenic and glycemic profile in polycystic ovary syndrome women undergoing in vitro fertilization treatment: A study protocol

**DOI:** 10.18502/ijrm.v21i5.13496

**Published:** 2023-05-12

**Authors:** Tayebeh Zohrabi, Mohammad Hasan Sheikhha, Sara Jambarsang, Azadeh Nadjarzadeh, Abbas Aflatoonian, Hassan Mozaffari-Khosravi

**Affiliations:** ^1^Department of Nutrition, School of Public Health, Shahid Sadoughi University of Medical Sciences, Yazd, Iran.; ^2^Nutrition and Food Security Research Center, Shahid Sadoughi University of Medical Sciences,Yazd, Iran.; ^3^Abortion Research Center, Yazd Reproductive Sciences Institute, Shahid Sadoughi University of Medical Sciences, Yazd, Iran.; ^4^Center for Healthcare Data Modeling, Departments of Biostatistics and Epidemiology, School of Public Health, Shahid Sadoughi University of Medical Sciences, Yazd, Iran.; ^5^Research and Clinical Center for Infertility, Yazd Reproductive Sciences Institute, Shahid Sadoughi University of Medical Sciences, Yazd, Iran.

**Keywords:** Polycystic ovary syndrome, Dietary approaches to stop hypertension, Curcumin, Fertilization in vitro, Gene expression.

## Abstract

**Background:**

Polycystic ovary syndrome (PCOS) is one of the most common endocrine diseases with major reproductive and metabolic complications with an impact on public health. Hyperandrogenism and chronic inflammation have been suggested as the leading cause of pathophysiology and clinical manifestations associated with PCOS. It seems that the altered expression of genes involved in the synthesis of pro-inflammatory cytokine and androgens contribute to the promotion of PCOS.

**Objective:**

This trial aims to determine the effects of dietary approaches to stop hypertension (DASH) and standard diets with and without curcumin supplementation on the gene expression of interleukin -1 alpha(IL1α), 5α reductase and androgenic and glycemic profile among PCOS patients, who are candidates for in vitro fertilization.

**Materials and Methods:**

96 infertile women with PCOS, aged 18-40 yr, will participate in this randomized, placebo-controlled clinical trial. Based on treatment conditions and body mass index, the participants will be randomly divided into 4 equal groups using a randomized block design. They will receive a DASH or standard diet containing 52% carbohydrate, 18% protein, and 30% total fat, with the same prescribed sodium, plus 500 mg twice daily curcumin or placebo for 12 wk. The mRNA expression of *IL-1 α *, *5 α* reductase, and androgenic and glycemic profiles will be measured at baseline and at the end of the study.

**Conclusion:**

Concomitant administration of DASH diet and curcumin supplementation may reduce *IL-1 α *, *5 α* reductase gene expressions, and improve glycemic and androgenic profiles.

## 1. Introduction

Polycystic ovary syndrome (PCOS) is an endocrine disorder that can lead to major reproductive and metabolic complications, including infertility and insulin resistance. According to the 2003 Rotterdam criteria, the prevalence of PCOS in women of reproductive age is 4.4-18.6% (1). The Rotterdam consensus is the most widely accepted diagnostic criteria (2), by which women must meet at least 2 of the 3 following criteria: 1) confirmation of polycystic ovary by ultrasound; 2) clinical or biochemical hyperandrogenism; and 3) ovulatory dysfunction (oligo/anovulation) (3).

Hyperandrogenism and chronic inflammation have been suggested as the leading causes of pathophysiology and clinical manifestations of PCOS (4). Biochemical hyperandrogenism is defined as a high concentration of testosterone and a high level of free testosterone and free androgen index (3). Hyperandrogenism may be caused by increased 5α-reductase activity, a key enzyme with an essential role in regulating the peripheral conversion of testosterone to the most active androgen, 5α-dihydrotestosterone (5). The findings showed that the activity of 5α-reductase increased in PCOS, and this high activity is related to insulin resistance (6). There are 2 isozymes of 5α reductase, encoded by the 5α-reductase type1 and type2 genes (7). Findings from an animal study suggest that insulin-mediated changes in 5α-reductase type1 messenger ribonucleic acid (mRNA) expression can negatively affect the follicle development and ovulation (8). In addition, mild chronic inflammation, independent of obesity, has been confirmed in PCOS patients (4). One of the most important inflammatory mediators, interleukin-1 (IL-1), is a multifunctional cytokine with 3 different cytokines, including cytokines (α, β) and an IL-1 receptor antagonist. *IL-1 α* gene polymorphism can be associated with PCOS and affect ovulation, fertilization, and implantation (9, 10).

Given the potential effects of chronic inflammation on the pathogenesis of PCOS through the pathway of insulin resistance and hyperandrogenism (11), new strategies have been proposed to improve the clinical symptoms of PCOS. According to evidence-based recommendations, lifestyle intervention (various components including diet, exercise, and behavioral approaches) is recommended (3).

The dietary approaches to stop hypertension (DASH) diet has been suggested as an effective treatment option (12). DASH diet, as a low-glycemic diet (13), with a high concentration of antioxidants, can have beneficial effects in reducing insulin resistance (14), inflammation (15), and free androgen index (16). This diet is rich in vegetables, low-fat dairy products, fruits, whole grains, poultry, fish, and nuts, but is limited in foods high in sodium, cholesterol, saturated fat, red and processed meat, refined grains, sweets, and sugary drinks (12). In the last decade, the antioxidative effects of plant polyphenols have been considered (17). Indeed, among natural phenols, the therapeutic properties of curcumin have been further investigated (18). Curcumin (diferuloylmethane) is a lipophilic yellow pigment extracted from turmeric rhizomes (*Curcuma longa L.*) (19). Curcumin supplementation improves glycemic control and lipid metabolism, and reduces radical oxygen species in PCOS patients (20, 21).

The anti-inflammatory and antioxidant benefits of both the DASH diet and curcumin have been demonstrated by meta-analysis results (15, 22). Therefore, we plan to investigate the effect of the DASH diet in combination with curcumin supplements on *IL-1 α* and *5 α* reductase gene expressions and improvement of insulin resistance and hyperandrogenism.

## 2. Materials and Methods

### Trial design and study setting

**Figure 1:**
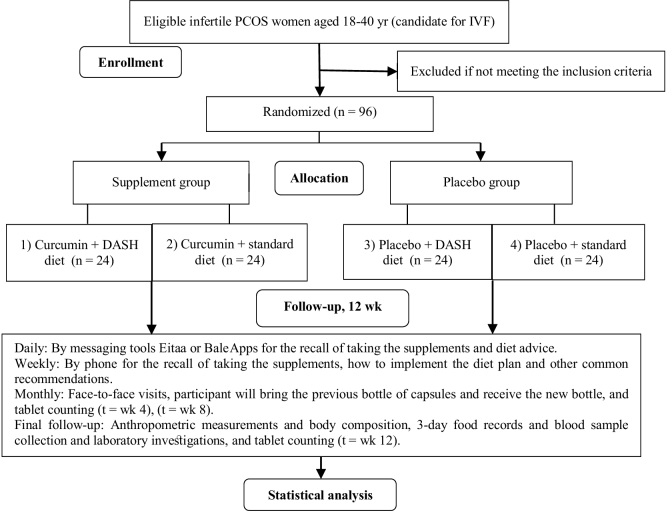
Overall overview of the study.

This randomized, placebo-controlled clinical trial will be conducted on PCOS women candidates for in vitro fertilization (IVF), using a convenience sampling method. Participants in a 2 × 2 factorial design will be randomly divided into 4 parallel treatment groups using block randomization: (1) curcumin supplement + DASH diet (2) curcumin supplement + standard diet (3) placebo + DASH diet, and (4) placebo + standard diet. The follow-up duration will be 12 wk. The study will be conducted at the Yazd Reproductive Sciences Institute, Shahid Sadoughi University of Medical Sciences, Yazd, Iran. An overview of the study is presented in figure 1.

### Eligibility criteria 

Infertile women with PCOS (according to the Rotterdam criteria) (2) who were candidates for IVF treatment, without a previous history of IVF, will be included in this study. Eligibility criteria included body mass index (BMI) between 18.5-25 and BMI ≥ 25 kg/m² and aged between 18–40 yr.

The participants must not have any of the following:

Metabolic and endocrine abnormalities including thyroid dysfunction, Cushing's syndrome, diabetes or impaired glucose tolerance, hyperprolactinemia, congenital adrenal hyperplasia, liver disease, cardiovascular disease, kidney disease, hypertension, neurological diseases, any history of mental illness, cancer, autoimmune and inflammatory disorders (rheumatoid arthritis). Taking medications including contraceptives or any other drugs that may affect reproductive physiology (during the 3 months before the study); taking antidiabetic, antiobesity, estrogenic, and androgenic drugs, as well as taking supplements such as vitamin C, vitamin E, and omega-3 because of their anti-inflammatory and antioxidative effects, 3 months before or during the intervention; smoking or exposure to spouse's smoking; alcohol drinking; being pregnant; simultaneous participation in another study. Moreover, unwillingness to cooperate and nonadherence to dietary or supplementary interventions (compliance < 80%), allergy to supplements, and pregnancy of the participant during the study will be considered as drop-out criteria.

### Interventions

Participants will be given 2 capsules of curcumin supplement per day (1 after breakfast and another after an evening meal with water) or 2 placebo capsules of curcumin per day at the same time for 12 wk. Curcumin capsules (BCM95/Curcugreen) and their placebos, with the same texture and appearance, were produced as 500 mg zero size veg capsules by “M/s Arjuna Natural Pvt Ltd., India”. The curcumin supplement was made from dried turmeric rhizomes by extraction with ethyl acetate. *Curcuma longa L. *commonly known as turmeric contains curcuminoids (curcumin, demethoxy curcumin, and bisdemethoxy curcumin) and essential oil of turmeric (ar-turmerone, curlone, and ar-curcumene). Curcuminoids are specific compounds with medicinal properties, of which the most important and active ingredient is the yellow coloring pigment curcumin; Ar-turmerone (the main bioactive components of the *Curcuma longa L*. essential oil), inhibits the P-glycoprotein pathway, thus improving the oral bioavailability and bioactivity of orally administered curcuminoids. BCM-95 was characterized by 95% total curcuminoid complex; the final formulation containing at least 65% curcumin and 86% total curcuminoids; and the essential oil containing not less than 45% ar-turmerone. Each 500-mg capsule contained 475 mg of curcuminoids and the essential oil of natural turmeric. Each placebo capsule contained 500 mg of roasted rice powder. The curcumin capsule actives will be analyzed by HPLC, heavy metals by ICP-MS; residual solvents by GCHS, pesticides by GCMS, and microbial indicators all of which were ensured to be in line with the EU standards (regulation (EC) No 1881/2006; directive 2009/32/CE; regulation (EC) No. 396/2005 and related amendments).

The intervention groups will receive 2 healthy dietary patterns (DASH or standard diet). These diets will be designed in the form of an individual diet containing 18% protein, 52% carbohydrates, and 30% total fat, which will be matched with the macronutrient plan used in the other original trial (23). Dietary programs will be planned after baseline assessments based on the current dietary intakes and body weight of each person. Daily calorie requirements will be estimated by calculating the resting energy expenditure and physical activity levels. If a person was overweight (BMI is 25.0 to < 30.0 kg/m²) or obese ( ≥ 30.0 kg/m²), the calculated energy will be reduced by 300 and 500 kcal, respectively. A 12-wk menu plan (7-day cycle menu with 21 meals) at 11 energy levels (1200, 1300, 1400, 1500, 1600, 1700, 1800, 1900, 2000, 2100, and 2200 kcal) for both dietary patterns will be constructed and analyzed. These will be considered as the basis for participants' diets. Nutritionist IV software (diet analysis, module version 3.5.2) will be used to calculate energy, food exchange, percent of kilocalories from macronutrients, and nutrient values in each daily menu plan. DASH diet will be designed with an emphasis on food rich in fiber, magnesium, potassium, calcium, and protein, such as vegetables, fruits, and low-fat dairy products; while foods high in saturated fat and sugar will be limited (12). The amount of sodium prescribed for each diet will be the same (2300 mg per day-approximately 1 teaspoon of salt). Overall, DASH and standard patterns will be designed using similar foods and recipes, but in different amounts, to achieve the specific characteristics of each diet. Both diets will be planned by the study dietitian and reviewed by the Chief investigator.

Participants will receive their specific diet through face-to-face individual counseling, initial assessment, and intervention for 45-60 min with a registered dietitian (follow-up visits for 15-30 min per month). They will also receive a bottle of supplement/placebo (on the 1 ${}^{\text{st}}$ day, 30 ${}^{\text{th}}$ day, and 60 ${}^{\text{th}}$ day of visit), each bottle containing 64 capsules (providing supplement for 1 month and a grace period of 2 days). Administration instructions on taking the pills and what needs to be done in case a dose is missed were set for patients, including taking 2 curcumin/placebo capsules a day (one after breakfast and one after an evening meal with water) and taking the missed capsule the same day. The patients will be monitored for daily supplementations by contacting them via phone interviews and messaging (Eitaa or Bale apps) twice a day. Moreover, daily consumption of supplements/placebos will be recorded in the case report form given to participants. At each follow-up visit, a nutritionist will assess the participants adherence to the diet and supplementation. The number of capsules taken will be calculated by counting the remaining capsules in each bottle. If the number of unused prescription capsules is more than 12 out of 60 capsules per month, for each participant, that patient will be classified in the non-adherent group.

All adverse events including unfavorable and/or unintended signs and any undesirable experience occurring to a patient will be reported in writing to the Medical Ethics Committee and the Data Monitoring Committee of the Shahid Sadoughi University of Medical Sciences, Yazd, Iran. The participants, for whatever reason, can discontinue participating in the research study at any given time before or after signing the consent form. The researcher may also terminate the study to maintain safety and protect the participant from excessive risks and/or to keep the integrity of the research data due to inappropriate follow-up by the participant. In case of termination of the study by the investigator, the reasons for the termination will be explained to the participants.

### Strategies to improve adherence to interventions

1) telephone follow-up; 2) weekly electronic counseling in order to improve patient's participation by reducing barriers such as geographical distance and time constraints; 3) husbands of the participant will also receive free dietary consulting to improve compliance and family support.

### Outcomes

The primary outcomes included gene expressions of IL-1α and 5α-reductase. The secondary outcomes consist serum levels of biochemical parameters comprising fasting blood sugar, fasting insulin, homeostasis model assessment of insulin resistance and quantitative insulin sensitivity check index, as well as serum androgen concentrations (testosterone, 5α-dihydrotestosterone) and anthropometric indices (height, weight, BMI, hip circumstance, waist circumstance, waist-hip ratio, waist-height ratio), body composition (muscle mass, fat mass, fat-free mass, and other factors such as trunk fat mass), physical activity, and dietary intake (energy and nutrient intake).

#### Demographic data and anthropometric measurements 

A form including: full address, age, gender, income, employment, ethnicity, level of education, and medical history (dose and type of medicine and supplements) will be completed for each participant.

Participant's weight, height, body circumferences (waist, hip), BMI, and waist-hip ratio as well as body composition analysis (fat mass, muscle mass, fat free mass) will be measured on the first and last day of the intervention. Weight will be measured to the nearest 0.5 kg (possible errors) using a pre-calibrated electronic scale (Seca, Germany). Height will be measured without shoes with an accuracy of 0.1 cm using a Seca wall stadiometer. Then BMI will be determined as weight (kg) divided by the square of their height (m²). Waist (cm) will be measured as the distance between the lowest rib and the iliac crest. Additionally, the hip circumference will be measured around the largest part of the hip. The waist-hip ratio will be the ratio of waist to hip circumference. The waist-height ratio will be measured by dividing the waist circumstance by the height. Body composition will be determined using body composition analyzer (Tanita BC-418, Tokyo, Japan), at the beginning of the study and after 12 wk of the study. All the measurements will be repeated by a trained assessor and recorded as average.

#### Biochemical measurements

After 2 wk of run-in period and considering 12-hr overnight fasting, 8 cc of blood samples will be collected in the morning from each participant, in the beginning, and at the end of the study.

5 cc of samples will be transferred to clot-activator tubes and then centrifuged for 30 min to separate the serum. Another 3 cc of blood samples will be collected in EDTA- coated tubes to seperate the plasma and buffy coat (the layer between red blood cells and plasma) and prepare buffy coat for gene expression assays. The allocated serum, plasma, and buffy coat will be stored immediately at -70 C. Before storage, the tripura isolation reagent will be added to the isolated buffy coat.

Serum levels of insulin, testosterone, and 5α-dihydrotestosterone will be evaluated using the enzyme-linked immunosorbent assay (ELIZA method), (ELISA kit, Monobind, California, United State). Fasting blood sugar will be measured by commercial kit using an enzymatic colorimetric method (Pars Azmoon, Tehran, Iran). Homeostasis model assessment of insulin resistance will be calculated, using the following formula: 


$$HOMA-IR=\frac{fastingbloodsugar\left(\frac{mg}{dl}\right)*Insulin}{450}$$

#### Gene expression assay

According to the kit protocol (Favorgen Biotech Corp., Taiwan), the buffy coat fraction of whole blood cells will be used for total RNA extraction. RNA concentration will be analyzed using spectrophotometer (DS-11, DeNovix) at 260 nm. Purity will be considered as an A260/A280 ratio of about 2 (24). cDNA will be synthesized from high-quality mRNA using a cDNA synthesis kit (GeneAll Biotechnology Co.). Forward and reverse primers are selected after reviewing previous studies (25-27) and checked by primer designing tools (Primer Blast, OLIGO 7 primer analysis software), as shown in table I.

IL-1α and 5α-reductase expression levels will be assessed by SYBR-green real-time PCR detection method using the kit protocol (Yekta Tajhiz Azma, IRAN), and the StepOne real-time PCR system (Applied Biosystems, Foster City, California, USA). GAPDH (glyceraldehyde phosphate dehydrogenase) will be chosen as the housekeeping gene.

**Table 1:** Real-time PCR primer sequences


**Gene**	**Forward**	**Reverse**
**5α-reductase **	TGGCGCTTCTCTATGGACTT	GGAAGCAACACTGCAGTTGA
**Interleukin 1 alpha **	ACTGCCCAAGATGAAGACCA	TTAGTGCCGTGAGTTTCCCA
***GAPDH *** **(Reference gene)**	CAAGAGCACAAGAGGAAGAGAGAG	TCTACATGGCAACTGTGAGGA
*GAPDH:* Glyceraldehyde-3-phosphate dehydrogenase, PCR: Polymerase chain reaction		

#### Dietary intake

Food consumption information will be obtained through 3-day dietary records including 2 days per week and one weekend. Before the study, participants will be given adequate training on food consumption and will be provided with a guide including written examples for completing a questionnaire that simply explains how to accurately record food. The participants will be taught to use standard kitchen measuring tools to accurately determine the portion size. To reduce memory errors, they will be asked to make a detailed list of foods and beverages exactly when eating. The expert dietitian will review the completed dietary records, ingredient lists, and preparation methods, while will be blind to the study allocation. Each daily food record will be calculated by the Nutritionist IV software.

#### Physical activity

The metabolic equivalent of task-hours (MET-h)/day will be applied to assess physical activity questionnaire data (28).

### Sample size

The sample size of 24 in each group was determined according to α = 0.05, the power of 80%, dropout rate of 20%, and considering a minimal clinically important difference of 0.969 and an effect size of 1, based on Heshmati et al. study (21) using the following formula: 


$$n=\frac{{\left(Z_{1-\alpha /2}+Z_{1-\beta }\right)}^{2}\left(S_{1}^{2}+S_{2}^{2}\right)}{{\left(\mu_{1}-\mu_{2}\right)}^{2}}$$

### Recruitment

Unique codes will be assigned to perform allocation concealment and prevent “selection bias". Supplements and placebos capsules will be provided in the same appearance (in terms of color and shape) and packed in similar bottles. The bottles will be labeled with different marks (A and B) and placed in envelopes with serial numbers by someone not related to the study. Participants and researchers will not be aware of the bottles content. Intervention groups will also receive diets from a nutritionist who is not involved in this study.

The participants will be assigned to 4 groups according to the method of stratified permuted block randomization based on treatment conditions and BMI (BMI: 18.5-25 and BMI ≥ 25), in equal block sizes of 4. The random allocation list will be created by Package `blockrand' in R software, version 4.0.2.

Randomization and allocation will be concealed from participants and researchers until finishing the study steps and data analysis. An independent biostatistician will generate the allocation sequence. 2 midwifes will give consecutive numbers to the eligible participants and refer them to a nutritionist. The sealed envelope containing the treatment code will be opened by the supervisor, after analyzing the final data or in an emergency condition.

All trial healthcare providers, data analysts, and outcome assessors will be blinded to intervention assignments. Participants may only be aware of the type of diet but will not be aware of the placebo or curcumin supplement content.

### Statistical methods

The values of quantitative variables will be presented as the mean ± standard deviation, and qualitative variables will be expressed as numbers and percentages. The normality of data will be examined by the Kolmogorov-Smirnov test.

The frequency among study groups will be compared using the Chi-square test. Independent sample *t* test and 2-way ANOVA will be applied to estimate the mean of quantitative variable changes and interactions between study groups; Bonferroni correction will be considered for multiple comparisons; and analysis of covariance (ANCOVA) will be used to compare the differences in the mean of quantitative data between the studied groups, while taking into account the influence of possible confounding variables. Before statistical analysis, all data will be checked for accuracy and completeness. All statistical analysis will be conducted using SPSS 22 (SPSS, Inc, Chicago, IL, USA). A p-value < 0.05 will be defined as a statistically significant level.

The “intention-to-treat" and “per protocol analysis" approaches will be performed for data analysis. The “intention-to-treat" analysis considers all participants in their main randomized groups, regardless of whether they follow the prescribed intervention according to the protocol or not, and also ignores non-compliance and withdrawal. Per protocol analysis, which accurately reflects what has been done, will be conducted based on actual adherence to the interventions.

### Participants and public involvement

Participants or the public will not participate in the setting, execution, measuring outcome, or dissemination plans of our study. The study results will be provided to patients upon request. Research findings will be reported in scientific journals.

### Ethical considerations

This study will be conducted following the Declaration of Helsinki. This trial was approved on September 2020 by the Medical Ethics Committee of Shahid Sadoughi University of Medical Sciences, Yazd, Iran, (Code: IR.SSU.SPH.REC.1399.145).

All goals, working methods, and possible side effects of the interventions will be explained to all the participants before starting and entering the study by the main researcher, and an informed consent form will be obtained from the participants.

Participants will be assured that all their information will be kept confidential. The contact information of one of the researchers will be provided to the participants for 24-hr contact in case of any complications or problems. The study will have no financial burden on the participants.

### Monitoring

Data monitoring and safety program are considered for this study. Project managers (HM, AA) and a research advisor (MS, SJ, and AN) will supervise and monitor the research process to ensure protocol compliance and data quality. Any withdrawals, lost to follow-up and adverse events will be assessed and reported. An independent trial Data Monitoring Committee will review the collected data and evaluate clinical trials for scientific validity, safety, and integrity.

### Study strengths and limitations

This will be the first study to investigate the effects of the DASH diet or curcumin supplementation on *IL-1 α* gene expression in human. This study will be the first clinical trial that investigates the co-administration of the DASH diet and curcumin supplementation in patients with PCOS. The frequency of PCOS women with BMI: 18.5-25 is probably low, which could increase study time and lead to bias in results.

##  Funding

This study is Ph.D. thesis and is funded by Shahid Sadoughi University of Medical Sciences, Yazd, Iran (grant no. 8229). This trial is externally supported by Iran National Science foundation (INSF) fund after review and approval by the INSF Scientific Committee (under Project No. 99019137 dated July 6, 2021).

##  Conflict of Interest

This funding source had no role in the design of this study and will not have any role during its execution, analyses, interpretation of the data, or decision to submit results. The authors have no conflict of interest to disclose.

##  Availability of data and materials

The datasets analyzed during the current study will be available from the corresponding author upon reasonable request.
